# Changes in the urinary proteome in rats with regular swimming exercise

**DOI:** 10.7717/peerj.12406

**Published:** 2021-11-01

**Authors:** Wenshu Meng, Dan Xu, Yunchen Meng, Weinan Zhang, Yaqi Xue, Zhiping Zhen, Youhe Gao

**Affiliations:** 1 Department of Biochemistry and Molecular Biology, Beijing Normal University, Gene Engineering Drug and Biotechnology Beijing Key Laboratory, Beijing, China; 2 College of P.E and Sports, Beijing Normal University, Beijing, China

**Keywords:** Urine, Proteome, Swimming, Exercise

## Abstract

**Purpose:**

Urine can sensitively reflect early pathophysiological changes in the body. The purpose of this study was to explore the changes of urine proteome in rats with regular swimming exercise.

**Methods:**

In this study, experimental rats were subjected to daily moderate-intensity swimming exercise for 7 weeks. Urine samples were collected at weeks 2, 5, and 7 and were analyzed by using liquid chromatography coupled with tandem mass spectrometry (LC-MS/MS).

**Results:**

Unsupervised clustering analysis of all urinary proteins identified at week 2 showed that the swimming group was distinctively different from the control group. Compared to the control group, a total of 112, 61 and 44 differential proteins were identified in the swimming group at weeks 2, 5 and 7, respectively. Randomized grouping statistical analysis showed that more than 85% of the differential proteins identified in this study were caused by swimming exercise rather than random allocation. According to the Human Protein Atlas, the differential proteins that have human orthologs were strongly expressed in the liver, kidney and intestine. Functional annotation analysis revealed that these differential proteins were involved in glucose metabolism and immunity-related pathways.

**Conclusion:**

Our results revealed that the urinary proteome could reflect significant changes after regular swimming exercise. These findings may provide an approach to monitor the effects of exercise of the body.

## Introduction

Urine is a good source for biomarker discovery. Without homeostatic mechanisms, urine can sensitively reflect early pathophysiological changes in the body, and these changes might be useful disease biomarkers (Gao, 2013). Since the composition of urine is affected by various factors, such as age, sex, and diet (Gao, 2014; Guo et al., 2015; Wu & Gao, 2015; Li & Gao, 2016), animal models are an effective tool to minimize external influencing factors due to their similar genetic backgrounds and the same living environment. Thus, disease animal models can establish relationships between the disease and the corresponding changes in the urine proteome. Our laboratory found that changes in urinary proteins occurred before pathologic or clinical manifestations appeared in various types of animal models, such as subcutaneous tumor model (Wu, Guo & Gao, 2017), Alzheimer’s disease model (Zhang et al., 2018b), chronic pancreatitis model (Zhang, Li & Gao, 2018), liver fibrosis model (Zhang et al., 2018a), and myocarditis model (Zhao et al., 2018). Recent studies have shown that the urine proteome has potential for differential diagnosis. For example, early urinary proteins were different when the same tumor cells were grown in different organs (Wu, Guo & Gao, 2017; Wei et al., 2019; Zhang et al., 2019; Wang et al., 2020; Zhang, Gao & Gao, 2020) and when different cells were injected into the same organ (Zhang et al., 2018a; Zhang et al., 2018b; Zhang et al., 2021). Furthermore, several clinical studies performed urine proteomics to discover diagnostic biomarkers, such as for gastric cancer (Shimura et al., 2020) and familial Parkinson’s disease (Winter et al., 2021).

Physical exercise as a pathophysiological process that can improve health conditions and has a positive role in numerous chronic conditions (Pate et al., 1995; Haskell et al., 2007; Seo et al., 2014), including cancer and coronary heart diseases (Stewart et al., 2017; Hojman et al., 2018). Many studies have shown that exercise has a profound effect on the immune system. Furthermore, it has been demonstrated that physical exercise exerts a positive impact on the nervous system, learning and memory (Inoue et al., 2015; Faria et al., 2016; Faria et al., 2018). Urinary proteomics of athletes after training and competition were analyzed in previous studies (Kohler et al., 2009; McCullough et al., 2011). To the best of our knowledge, there are very few studies on global urinary proteomes after daily exercise. Swimming is a popular physical activity and an effective option for maintaining and improving cardiovascular health. Recent studies have shown that swimming is beneficial for mental health and cognitive ability (Hillman, Castelli & Buck, 2005; Da Silva et al., 2020). Rats have the innate ability to swim and are the first choice for swimming models (Souza et al., 2009). The purpose of this study was to explore the changes of urine proteome in rats with regular swimming exercise.

**Figure 1:**
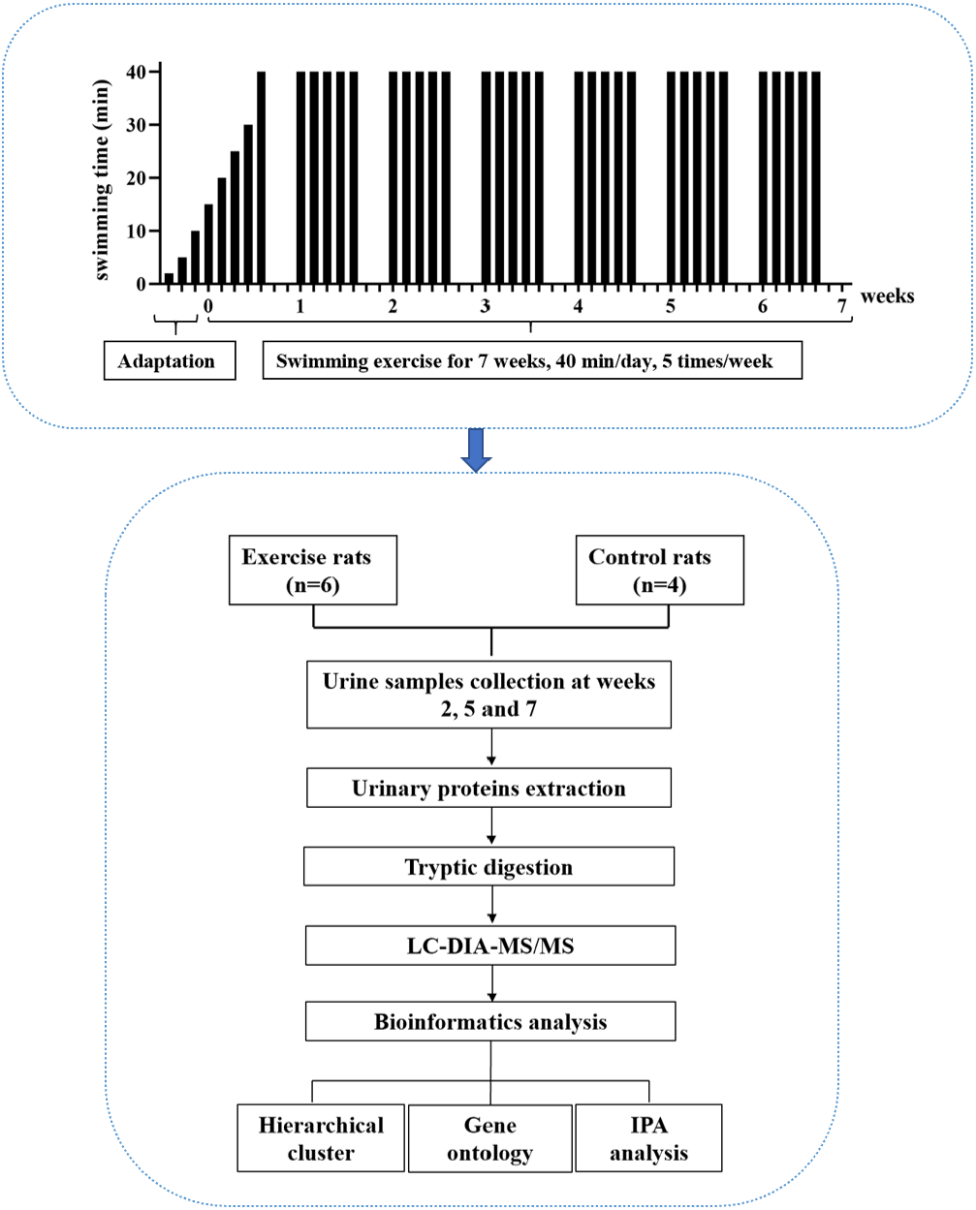
The experimental design and workflow of the proteomics analysis in this study. The experimental rats were subjected to daily moderate-intensity swimming exercise for 7 weeks. Urine samples were collected at weeks 2, 5, and 7 during swimming exercise. Urine proteins were identified by liquid chromatography coupled with tandem mass spectrometry (LC-MS/MS).

In this study, experimental rats were subjected to daily moderate-intensity swimming exercise for 40 min per day for 7 weeks. Urine samples were collected at weeks 2, 5, and 7. The urine proteome was analyzed by liquid chromatography coupled with tandem mass spectrometry (LC-MS/MS). The experimental design and workflow of the proteomics analysis in this study are shown in Fig. 1.

## Materials & Methods

### Experimental animals

Male SD rats (seven days old) were supplied by the Department of Neurobiology, School of Basic Medical Sciences, Peking University. All animals were housed with free access to a standard laboratory diet and water with a 12-h light-dark cycle under standard conditions (indoor temperature 22 ± 1 °C, humidity 65–70%). The experiment was approved by the Institute of Basic Medical College (ID: ACUC-A02-2014-007). The study was performed according to guidelines developed by the Institutional Animal Care and Use Committee of Peking Medical College. After the experiment, all the animals were euthanized by intraperitoneal injection of barbiturates.

### Swimming exercise

A large pool (diameter: 1,500 mm, height: 500 mm) served as the swimming pool. The water temperature was maintained at 36 °C. For the adaptation phase, rats swam for increasing amounts of time, from 2 min to 10 min over three days. For the exercise phase, the intensity of exercise in the first week gradually increased from 15 min to 40 min, and the intensity at 40 min lasted for 6 weeks, which is considered to be moderate exercise (Seo et al., 2014). The animals were quickly and gently dried after each training session. The rats (*n* = 10) were randomly divided into the following two groups: experimental rats (*n* = 6) and control rats (*n* = 4). In the experimental group, the rats underwent the 7-week swimming exercise program. The control rats did not swim.

### Urine collection and sample preparation

Urine samples were collected from the experimental and control groups at weeks 2, 5 and 7 during the swimming exercise. The animals were individually placed in metabolic cages for 10 h to collect urine samples without any treatment. After collection, the urine samples were stored at −80 °C. The urine samples (*n* = 30) were centrifuged at 12,000 g for 40 min at 4 °C to remove cell debris. The supernatants were precipitated with three volumes of ethanol at −20 °C overnight and then centrifuged at 12,000 g for 30 min. Then, lysis buffer (8 mol/L urea, 2 mol/L thiourea, 50 mmol/L Tris, and 25 mmol/L DTT) was used to dissolve the pellets. The protein concentration of the urine samples was measured by the Bradford assay.

### Tryptic digestion

Urinary proteins (100 µg of each sample) were digested with trypsin (Trypsin Gold, Mass Spec Grade, Promega, Fitchburg, WI, USA) using filter-aided sample preparation (FASP) methods (Wisniewski et al., 2009). These peptide mixtures were desalted using Oasis HLB cartridges (Waters, Milford, MA) and dried by vacuum evaporation (Thermo Fisher Scientific, Bremen, Germany). The digested peptides (*n* = 30) were redissolved in 0.1% formic acid to a concentration of 0.5 µg/µL. The iRT reagent (Biognosys, Switzerland) was spiked at a concentration of 1:10 v/v into all samples for calibration of the retention time of the extracted peptide peaks. For analysis, 1 µg of peptides from an individual sample was analyzed by LC-DIA-MS/MS.

### Reversed-phase fractionation spin column separation

A total of 90 µg of pooled peptides was generated from 6 µl from each sample and then separated by a high-pH reversed-phase peptide fractionation kit (Thermo Pierce, Waltham, MA, USA) according to the manufacturer’s instructions. A step gradient of increasing acetonitrile concentrations (5, 7.5, 10, 12.5, 15, 17.5, 20 and 50%) was applied to the columns to elute the peptides. Ten different fractionated samples (including the flow-through fraction, wash fraction, and eight step gradient sample fractions) were collected and dried by vacuum evaporation. The ten fractions were resuspended in 20 µl of 0.1% formic acid, and 1 µg of each of the fractions was analyzed by LC-DDA-MS/MS.

### LC-MS/MS analysis

A total of 30 peptide samples were analyzed in an EASY-nLC 1200 chromatography system (Thermo Fisher Scientific) and an Orbitrap Fusion Lumos Tribrid mass spectrometer (Thermo Fisher Scientific). The samples were loaded onto a trapping column (75 µm × 2 cm, 3 µm, C18, 100 Å) and separated by a reverse-phase analysis column (75 µm × 25 cm, 2 µm, C18, 100 Å). The eluted gradient was 4%–35% buffer B (0.1% formic acid in 80% acetonitrile) at a flow rate of 300 nL/min for 90 min.

To generate the spectral library, 1 µg of each of ten fractions was analyzed in DDA mode. The parameters were set as follows: the full scan ranged from 350 to 1500 m/z with a resolution of 120,000; MS/MS scans were acquired with a resolution of 30,000; the cycle time was set to 3 s; the HCD energy was set to 30%; the autogain control (AGC) target was set to 4e5; and the maximum injection time was set to 50 ms. In DIA mode, 1 µg of each sample was analyzed. The variable isolation window of the DIA method with 36 windows was set (Table S1). The parameters were set as follows: the full scan ranged from 350 to 1,500 m/z with a resolution of 60,000; the DIA scan was acquired from 200 to 2,000 m/z with a resolution of 30,000; the HCD energy was set to 32%; the AGC target was set to 1e6; and the maximum injection time was set to 100 ms. During the samples analysis, a mixture from each sample was analyzed after every six samples for quality control (QC).

### Data analysis

The DDA data of ten fractions were searched against the Swiss-Prot rat database (released in 2017, including 7,992 sequences) appended with the iRT peptide sequence using Proteome Discoverer software (version 2.1, Thermo Scientific). The search parameters were set as follows: two missed trypsin cleavage sites were allowed; the parent ion mass tolerances were set to 10 ppm; the fragment ion mass tolerances were set to 0.02 Da; the carbamidomethyl of cysteine was set as a fixed modification; and the oxidation of methionine was set as a variable modification. The false discovery rate (FDR) of the proteins was less than 1%. A total of 873 protein groups, 4098 peptide groups and 37555 peptide spectrum matches were identified. The search results were used to set the DIA method. The DDA raw files were processed using Spectronaut’s Pulsar database (Biognosys, Switzerland) with the default parameters to generate the spectral library. The DIA raw files were processed using Spectronaut for analysis with the default setting. All of the results were filtered by a Q value cutoff of 0.01 (corresponding to an FDR of 1%). Peptide intensity was calculated by summing the peak areas of their respective fragment ions of MS2, and the protein intensity was calculated by summing the intensities of their respective peptides.

### Statistical analysis

The k-nearest neighbor (K-NN) method was used to fill the missing values of protein abundance (Armitage et al., 2015). Comparisons between experimental and control groups were performed by one-way ANOVA. The differential proteins at weeks 2, 5 and 7 were screened by the following criteria: fold change ≥ 1.5 or ≤ 0.67; and *P* < 0.05 by independent sample *t*-test. Group differences resulting in *p* < 0.05 were considered statistically significant.

### Functional annotation of the differential proteins

DAVID 6.8 (https://david.ncifcrf.gov/) was used to perform the functional annotation of the differential proteins between the experimental and control groups. The canonical pathways were analyzed with IPA software (Ingenuity Systems, Mountain View, CA, USA).

## Results

### Urine proteome changes in the swimming exercise rats

In this study, thirty urine samples from three time points (weeks 2, 5, and 7) from six experimental rats and four control rats were used for LC-DIA-MS/MS quantitation. A total of 729 proteins and 5,265 peptides were identified in all urine samples. A quality control sample of a mixture from each sample was analyzed after every six samples. A total of 518 proteins were identified that had a coefficient of variation (CV) of the QC samples below 30%, and all of the identification and quantification details are listed in Table S2.

**Figure 2:**
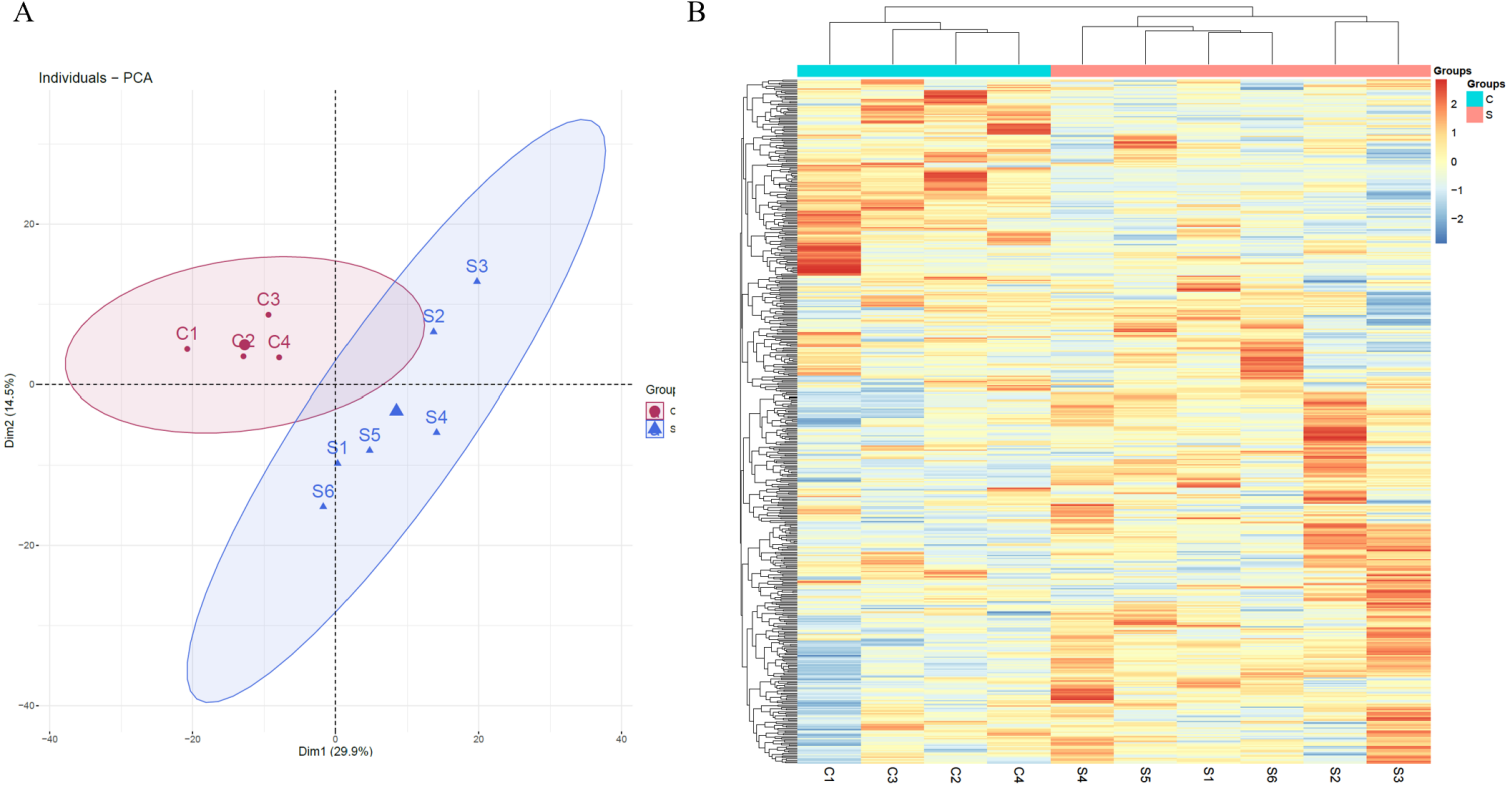
Proteomic analysis of the urine samples of swimming exercise rats. (A) PCA analysis of all proteins from experimental and control urine proteome at week 2. (B) Cluster analysis of all the proteins from experimental and control urine proteome at week 2.

Unsupervised clustering analysis of all of proteins identified at three time points was performed (Fig. S1). We found that the samples at week 2 were clustered together, indicating that swimming exercise has a great impact on urine after 2 weeks. It is speculated that the clustering effect of the samples at weeks 5 and 7 was poor because the body had adapted to long-term exercise. To further characterize the effects of 2 weeks of swimming exercise, all urinary proteins from 10 urine samples between the two groups at week 2 were analyzed by principal component analysis (PCA). As shown in Fig. 2A, the swimming exercise rats were differentiated from the control rats. Meanwhile, unsupervised clustering analysis of all urinary proteins from 10 urine samples between two groups at week 2 was performed. As shown in Fig. 2B, the proteomics profiles of the swimming group were distinctively different from those of the control group. These results demonstrated that the urinary proteome changed significantly after swimming exercise.

**Figure 3:**
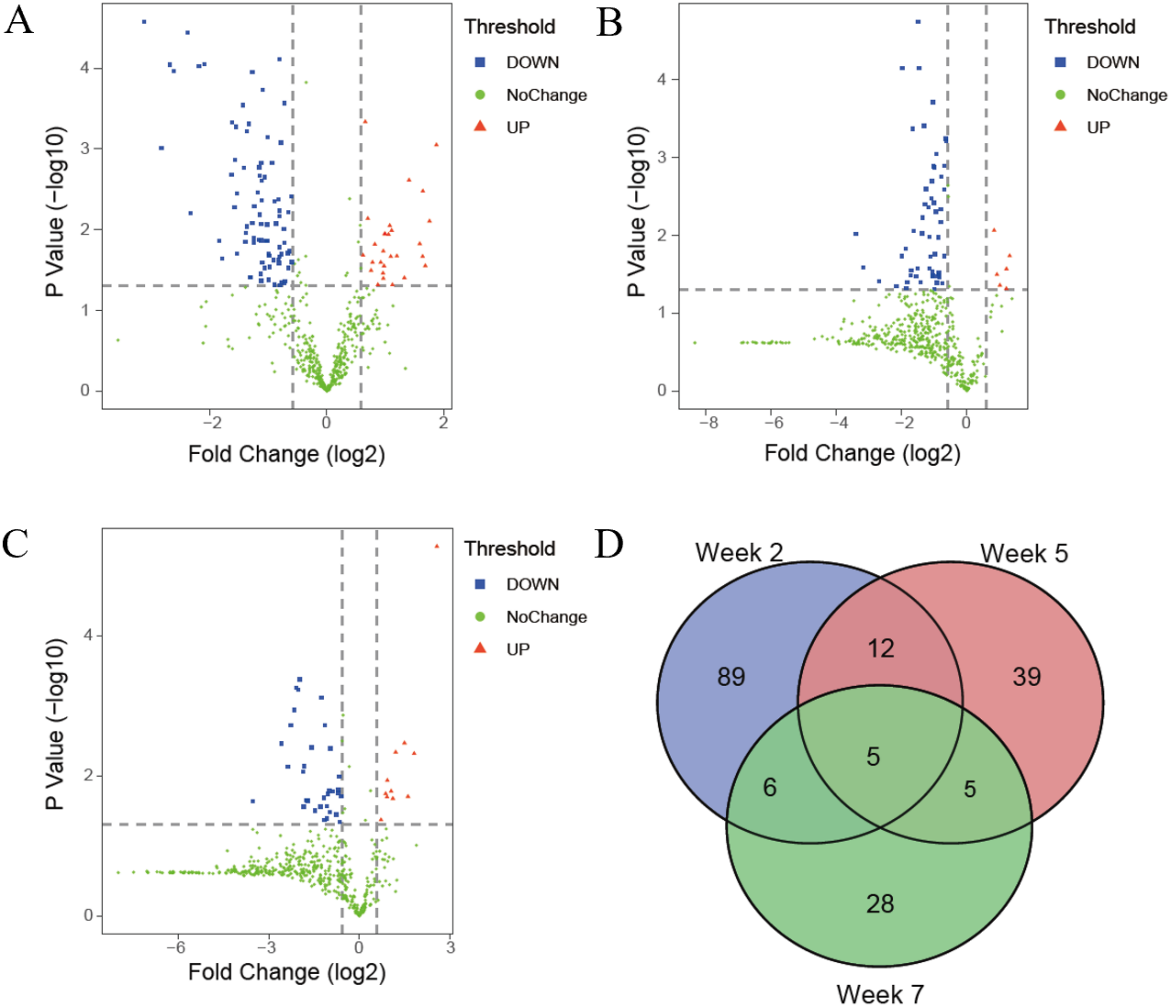
Differential proteins identified between experimental and control group. (A) Volcano plots showing *P* values (−log10) *versus* protein ratios between experimental and control rats (log2) at week 2. (B) Volcano plots showing *P* values (−log10) *versus* protein ratios between experimental and control rats (log2) at week 5. (C) Volcano plots showing *P* values (−log10) *versus* protein ratios between experimental and control rats (log2) at week 7. (D) Overlap evaluation of differential proteins at three time points.

The differential proteins were screened with a *p* value < 0.05 by two-sided, unpaired *t*-test and a fold change ≥ 1.5 compared with controls. Compared to the control group, 112 differential proteins were identified after 2 weeks of swimming exercise, among which 28 proteins were upregulated and 84 proteins were downregulated (Fig. 3A); 61 differential proteins were identified after 5 weeks of swimming exercise, among which 6 proteins were upregulated and 55 proteins were downregulated (Fig. 3B); and 44 differential proteins were identified after 7 weeks of swimming exercise, among which 11 proteins were upregulated and 33 proteins were downregulated (Fig. 3C). The details of these differential proteins are presented in Table 1. Among these differential proteins, 171 proteins had human orthologs. The overlap of these differential proteins is shown by the Venn diagram in Fig. 3D. Five proteins were commonly identified at three time points (Fig. 3D), including Ig gamma-1 chain C region, hemopexin, transthyretin, cathepsin D and chondroitin sulfate proteoglycan 4.

**Table 1:** The details of differential proteins identified at three time points.

**Uniprot ID**	**Protein names**	**Human ortholog**	***P*-value**	**Fold change**		
			**Week 2**	**Week 5**	**Week 7**
P20059	Hemopexin	P02790	0.039679	2.501	1.896	3.514
P24268	Cathepsin D	P07339	0.000272	0.605	0.420	0.453
P02767	Transthyretin	P02766	0.049310	0.575	0.316	0.618
P20759	Ig gamma-1 chain C region	P01859	0.003593	0.346	0.322	0.483
Q00657	Chondroitin sulfate proteoglycan 4	Q6UVK1	0.000090	0.235	0.254	0.419
Q63556	Serine protease inhibitor A3M	P01011	0.021564	3.103	0.479	
P27590	Uromodulin	P07911	0.002444	2.642	0.453	
Q6IFW6	Keratin, type I cytoskeletal 10	P13645	0.048471	2.172	2.019	
O70534	Protein delta homolog 1	P80370	0.048375	1.828	0.463	
P82450	Sialate O-acetylesterase	Q9HAT2	0.007297	1.623	0.647	
P47820	Angiotensin-converting enzyme	P12821	0.018595	0.642	0.303	
Q6AYS7	Aminoacylase-1A	Q03154	0.023841	0.532	2.322	
P02651	Apolipoprotein A-IV	P06727	0.029010	0.508	0.362	
P13635	Ceruloplasmin	P00450	0.002251	0.479	0.501	
Q64319	Neutral and basic amino acid transport protein rBAT	Q07837	0.025707	0.465	2.484	
B5DFC9	Nidogen-2	Q14112	0.005150	0.423	0.392	
P20761	Ig gamma-2B chain C region	NO	0.001365	0.338	1.785	
P00689	Pancreatic alpha-amylase	NO	0.015215	1.763		5.916
P29975	Aquaporin-1	P29972	0.016488	0.569		0.334
P52759	2-iminobutanoate/2-iminopropanoate deaminase	P52758	0.006852	0.569		0.589
P08721	Osteopontin	P10451	0.048467	0.541		0.247
O70513	Galectin-3-binding protein	Q08380	0.000185	0.468		0.507
P01015	Angiotensinogen	P01019	0.003462	0.440		2.158
P14046	Alpha-1-inhibitor 3	NO	0.040909		0.529	0.446
P10960	Prosaposin	P07602	0.001299		0.491	0.449
Q99041	Protein-glutamine gamma-glutamyltransferase 4	P49221	0.028307		0.306	0.225
Q4V885	Collectin-12	Q5KU26	0.014972		0.273	0.305
P05544	Serine protease inhibitor A3L	P01011	0.000894	3.651		
P28648	CD63 antigen	P08962	0.007863	3.366		
P84039	Ectonucleotide pyrophosphatase/phosphodiesterase family member 5	Q9UJA9	0.028089	3.202		
P05545	Serine protease inhibitor A3K	P01011	0.003344	3.111		
O89117	Beta-defensin 1	P60022	0.015008	2.999		
P32038	Complement factor D	P00746	0.021309	2.288		
P15950	Glandular kallikrein-3, submandibular	NO	0.010257	2.152		
P20909	Collagen alpha-1	P12107	0.021608	2.129		
Q63514	C4b-binding protein alpha chain	P04003	0.008936	2.104		
D3ZTX0	Transmembrane emp24 domain-containing protein 7	Q9Y3B3	0.011501	2.073		
P49134	Integrin beta-1	P05556	0.011293	1.988		
Q76HN1	Hyaluronidase-1	Q12794	0.011380	1.982		
Q6RY07	Acidic mammalian chitinase	Q9BZP6	0.028264	1.965		
P05539	Collagen alpha-1	P02458	0.040399	1.950		
Q6AXR4	Beta-hexosaminidase subunit beta	P07686	0.018512	1.949		
Q9R1T3	Cathepsin Z	Q9UBR2	0.034738	1.942		
Q5XIL0	E3 ubiquitin-protein ligase RNF167	Q9H6Y7	0.025554	1.891		
P48199	C-reactive protein	P02741	0.025494	1.716		
P08649	Complement C4	P0C0L4	0.032173	1.686		
P50430	Arylsulfatase B	P15848	0.000462	1.575		
Q6AYP5	Cell adhesion molecule 1	Q9BY67	0.020851	1.534		
Q00238	Intercellular adhesion molecule 1	P05362	0.025199	0.662		
Q920H8	Hephaestin	Q9BQS7	0.003917	0.659		
B0BND0	Glycerophosphocholine cholinephosphodiesterase ENPP6	Q6UWR7	0.030004	0.636		
Q6Q0N1	Cytosolic non-specific dipeptidase	Q96KP4	0.006146	0.636		
P29598	Urokinase-type plasminogen activator	P00749	0.019588	0.630		
Q8R5M3	Leucine-rich repeat-containing protein 15	Q8TF66	0.043831	0.608		
Q62638	Golgi apparatus protein 1	Q92896	0.014649	0.607		
P31044	Phosphatidylethanolamine-binding protein 1	NO	0.046012	0.606		
Q9QX79	Fetuin-B	Q9UGM5	0.009853	0.602		
Q5U367	Multifunctional procollagen lysine hydroxylase and glycosyltransferase LH3	O60568	0.020139	0.586		
Q5U2Q3	Ester hydrolase C11orf54 homolog	Q9H0W9	0.045189	0.584		
P35704	Peroxiredoxin-2	P32119	0.000841	0.581		
P46413	Glutathione synthetase	P48637	0.021466	0.573		
Q4FZV0	Beta-mannosidase	O00462	0.000078	0.571		
Q99MA2	Xaa-Pro aminopeptidase 2	O43895	0.005837	0.569		
Q63530	Phosphotriesterase-related protein	Q96BW5	0.026074	0.567		
P48500	Triosephosphate isomerase	P60174	0.004231	0.566		
P19804	Nucleoside diphosphate kinase B	P22392	0.023348	0.564		
P27139	Carbonic anhydrase 2	P00918	0.008439	0.548		
P51647	Retinal dehydrogenase 1	P00352	0.004435	0.538		
P04639	Apolipoprotein A-I	P02647	0.001486	0.524		
P51635	Aldo-keto reductase family 1 member A1	P14550	0.021157	0.509		
P69897	Tubulin beta-5 chain	P07437	0.019827	0.504		
P53813	Vitamin K-dependent protein S	P07225	0.021142	0.503		
P62963	Profilin-1	P07737	0.042120	0.501		
P08650	Complement C5	NO	0.000712	0.496		
Q9QXQ0	Alpha-actinin-4	O43707	0.013840	0.494		
O55004	Ribonuclease 4	P34096	0.008646	0.492		
P85971	6-phosphogluconolactonase	O95336	0.013814	0.488		
P19112	Fructose-1,6-bisphosphatase 1	P09467	0.029793	0.480		
Q62930	Complement component C9	P02748	0.004371	0.464		
P60711	Actin, cytoplasmic 1	P60709	0.002492	0.462		
P22282	Cystatin-related protein 1	NO	0.043085	0.460		
P42123	L-lactate dehydrogenase B chain	P07195	0.001504	0.460		
P08289	Alkaline phosphatase, tissue-nonspecific isozyme	P05186	0.013575	0.460		
P05964	Protein S100-A6	P06703	0.008557	0.459		
P00884	Fructose-bisphosphate aldolase B	P05062	0.035549	0.457		
P50399	Rab GDP dissociation inhibitor beta	P50395	0.006541	0.453		
P02770	Albumin	P02768	0.002136	0.451		
Q63716	Peroxiredoxin-1	Q06830	0.001691	0.449		
Q06496	Sodium-dependent phosphate transport protein 2A	Q06495	0.013307	0.448		
P41562	Isocitrate dehydrogenase [NADP] cytoplasmic	O75874	0.014681	0.422		
P01041	Cystatin-B	P04080	0.012801	0.421		
P04642	L-lactate dehydrogenase A chain	P00338	0.000113	0.415		
Q9Z339	Glutathione S-transferase omega-1	P78417	0.008351	0.415		
D4ACX8	Protocadherin-16	Q96JQ0	0.039161	0.408		
Q9WTW7	Solute carrier family 23 member 1	Q9UHI7	0.038746	0.402		
P17475	Alpha-1-antiproteinase	P01009	0.000491	0.396		
P50115	Protein S100-A8	P05109	0.009132	0.389		
Q6P734	Plasma protease C1 inhibitor	P05155	0.000603	0.388		
P34080	Aquaporin-2	P41181	0.010950	0.383		
P20762	Ig gamma-2C chain C region	NO	0.014244	0.382		
Q5FVQ0	Metal cation symporter ZIP8	Q9C0K1	0.001720	0.376		
P15978	Class I histocompatibility antigen, Non-RT1.A alpha-1 chain	P01891	0.000287	0.370		
P34058	Heat shock protein HSP 90-beta	P08238	0.019878	0.344		
P09006	Serine protease inhibitor A3N	P01011	0.000536	0.341		
P04276	Vitamin D-binding protein	P02774	0.005304	0.335		
Q66HG4	Galactose mutarotase	Q96C23	0.000467	0.326		
Q63772	Growth arrest-specific protein 6	Q14393	0.002087	0.323		
P50116	Protein S100-A9	P06702	0.022965	0.290		
P00697	Lysozyme C-1	P61626	0.013674	0.279		
P12346	Serotransferrin	P02787	0.000095	0.220		
P01026	Complement C3	P01024	0.006223	0.200		
P06866	Haptoglobin	P00739	0.000036	0.193		
Q64268	Heparin cofactor 2	P05546	0.000109	0.164		
Q9EQV9	Carboxypeptidase B2	Q96IY4	0.000091	0.156		
Q63313	Lipopolysaccharide-binding protein	P18428	0.000977	0.141		
P15399	Probasin	NO	0.000027	0.115		
Q6IFV1	Keratin, type I cytoskeletal 14	P02533	0.048522		2.314	
Q642A7	Protein FAM151A	Q8WW52	0.000571		0.641	
P23680	Serum amyloid P-component	P02743	0.001278		0.622	
P70490	Lactadherin	Q08431	0.002590		0.619	
P15083	Polymeric immunoglobulin receptor	P01833	0.018502		0.617	
P16391	RT1 class I histocompatibility antigen, AA alpha chain	NO	0.041460		0.590	
P36373	Glandular kallikrein-7, submandibular/renal	P06870	0.001770		0.582	
Q05820	Putative lysozyme C-2	P61626	0.006730		0.575	
Q63041	Alpha-1-macroglobulin	NO	0.004656		0.574	
P26051	CD44 antigen	P16070	0.034146		0.560	
P61972	Nuclear transport factor 2	P61970	0.029915		0.550	
Q63621	Interleukin-1 receptor accessory protein	Q9NPH3	0.031691		0.546	
P97829	Leukocyte surface antigen CD47	Q08722	0.010609		0.543	
Q6AYE5	Out at first protein homolog	Q86UD1	0.033694		0.541	
P13221	Aspartate aminotransferase, cytoplasmic	P17174	0.000894		0.521	
P54759	Ephrin type-A receptor 7	Q15375	0.004971		0.518	
P16573	Carcinoembryonic antigen-related cell adhesion molecule 1	P13688	0.017399		0.506	
P36376	Glandular kallikrein-12, submandibular/renal	P06870	0.049426		0.500	
Q9WUK5	Inhibin beta C chain	P55103	0.003809		0.497	
Q9EPB1	Dipeptidyl peptidase 2	Q9UHL4	0.000194		0.486	
Q9R0D6	Transcobalamin-2	P20062	0.033115		0.484	
P13852	Major prion protein	P04156	0.033316		0.479	
P43303	Interleukin-1 receptor type 2	P27930	0.002016		0.474	
Q9R0T4	Cadherin-1	P12830	0.003331		0.473	
Q794F9	4F2 cell–surface antigen heavy chain	P08195	0.026565		0.450	
Q64604	Receptor-type tyrosine-protein phosphatase F	P10586	0.004355		0.444	
Q6IUU3	Sulfhydryl oxidase 1	O00391	0.003978		0.412	
Q7TPB4	CD276 antigen	Q5ZPR3	0.000390		0.403	
P11232	Thioredoxin	P10599	0.009423		0.386	
P98158	Low-density lipoprotein receptor-related protein 2	P98164	0.040045		0.373	
P53369	7,8-dihydro-8-oxoguanine triphosphatase	P36639	0.000018		0.354	
P35859	Insulin-like growth factor-binding protein complex acid labile subunit	P35858	0.026722		0.351	
P07154	Procathepsin L	P07711	0.033555		0.341	
Q91XT9	Neutral ceramidase	Q9NR71	0.039975		0.281	
Q0PMD2	Anthrax toxin receptor 1	Q9H6X2	0.048291		0.276	
Q30 kJ2	Beta-defensin 50		0.018575		0.251	
P52796	Ephrin-B1	P98172	0.045230		0.224	
P32736	Opioid-binding protein/cell adhesion molecule	Q14982	0.038970		0.156	
P97580	Beta-microseminoprotein	P08118	0.025914		0.110	
P06760	Beta-glucuronidase	P08236	0.009520		0.095	0.208
P20760	Ig gamma-2A chain C region	P01859	0.019815			3.050
P42854	Regenerating islet-derived protein 3-gamma	NO	0.003409			2.815
P01836	Ig kappa chain C region, A allele	P01834	0.004604			2.299
P20611	Lysosomal acid phosphatase	P11117	0.016619			2.073
P01835	Ig kappa chain C region, B allele	P01834	0.011652			1.900
Q920A6	Retinoid-inducible serine carboxypeptidase	Q9HB40	0.020110			1.885
Q5XI43	Matrix remodeling-associated protein 8	Q9BRK3	0.018083			1.828
Q6P7S1	Acid ceramidase	Q13510	0.042780			1.643
Q499T2	Gamma-interferon-inducible lysosomal thiol reductase	P13284	0.019485			0.660
P08592	Amyloid-beta A4 protein	P05067	0.045641			0.637
P07897	Aggrecan core protein	P16112	0.010204			0.629
Q9JHY1	Junctional adhesion molecule A	Q9Y624	0.016015			0.615
P04906	Glutathione S-transferase P	P09211	0.016839			0.541
Q6MG71	Choline transporter-like protein 4	Q53GD3	0.004089			0.515
P0CG51	Polyubiquitin-B [Cleaved into: Ubiquitin]	P0CG47	0.016453			0.504
P02650	Apolipoprotein E	P02649	0.040693			0.477
Q6TUD4	Protein YIPF3	Q9GZM5	0.027382			0.472
Q05695	Neural cell adhesion molecule L1	P32004	0.027649			0.411
P10247	H-2 class II histocompatibility antigen gamma chain	P04233	0.031337			0.362
Q9JJ19	Na(+)/H(+) exchange regulatory cofactor NHE-RF1	O14745	0.022332			0.297
Q62632	Follistatin-related protein 1	Q12841	0.007274			0.282
Q5M871	Fas apoptotic inhibitory molecule 3	O60667	0.027711			0.279
Q9WUC4	Copper transport protein ATOX1	O00244	0.008730			0.275
Q06880	Neuroblastoma suppressor of tumorigenicity 1	P41271	0.000421			0.254
Q63467	Trefoil factor 1	P04155	0.000552			0.234
P07171	Calbindin	P05937	0.007365			0.193
Q09030	Trefoil factor 2	Q03403	0.003471			0.168
P97574	Stanniocalcin-1	P52823	0.023304			0.087

### Tissue distribution of the human orthologs of the differential proteins

**Figure 4:**
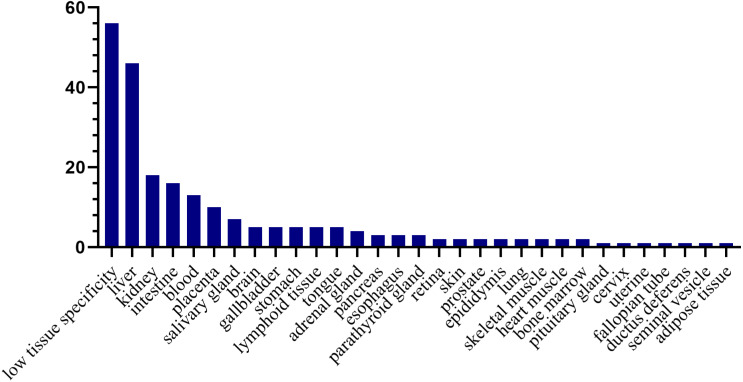
Tissue distribution of the human orthologs of differential proteins. X-axis represents human tissues; Y- axis represents the number of differential proteins.

To investigate the expression levels of the differential proteins in different tissues and organs, 171 differential proteins that had human orthologs were searched from the Human Protein Atlas. According to the Tissue Atlas, 31 tissues were identified (Fig. 4). The differential proteins were strongly expressed in the liver, kidney, intestine, and blood, indicating that these organs may be affected after swimming exercise. Swimming exercise can recruit a large volume of muscle mass. Notably, two proteins, triosephosphate isomerase (TIPSS) and aspartate aminotransferase (AATC), were strongly expressed in skeletal muscle, indicating that moderate-intensity swimming exercise might have an effect on the muscles of rats.

### Randomized grouping statistical analysis

Considering that omics data are large but the sample size is limited, the differences between the two groups may be randomly generated. To confirm whether the differential proteins were indeed due to swimming exercise, we performed a randomized grouping statistical analysis. We randomly allocated the proteomic data of 10 samples (6 for experimental and 4 for control samples) at each time point and screened for the differential proteins with the same criteria. Then, the average number of differential proteins in all random combinations was calculated, which was the false positive in the actual grouping. There were 210 random allocations at each time point, and the average number of differential proteins in all random combinations at each time point was 15, 5 and 6. The results showed that the false-positive rates were 13.4%, 5% and 13.6% at weeks 2, 5 and 7, respectively. Therefore, most of the differential proteins identified at each time point in this study were caused by swimming exercise rather than random allocation. The details are presented in Table S3. These results suggested that the sample size of this study was sufficient to prove the significant difference in the urine proteome between the swimming group and the control group.

### Functional annotation analysis of the differential proteins

Functional annotation of differential proteins at weeks 2, 5 and 7 was performed by DAVID (Huang, Sherman & Lempicki, 2009). The differential proteins identified at three time points were classified into three categories: biological process, cellular component and molecular function.

**Figure 5:**
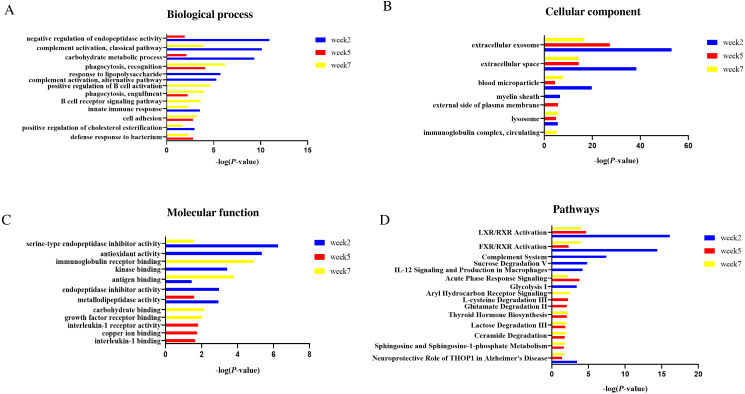
Functional enrichment analysis of differential proteins in this study. (A) Biological process (B) Cellular component (C) Molecular function (D) Canonical pathways.

In the biological process category (Fig. 5A), negative regulation of endopeptidase activity and carbohydrate metabolic process were overrepresented at weeks 2 and 5; complement activation, classical pathway, innate immune response and positive regulation of cholesterol esterification were overrepresented at weeks 2 and 7. Response to lipopolysaccharide and positive regulation of cholesterol esterification were only overrepresented at week 2; B cell receptor signaling pathway and positive regulation of B cell activation were only overrepresented at week 7.

In the cellular component category (Fig. 5B), the majority of these differential proteins were from extracellular exosome and extracellular space. In the molecular function category (Fig. 5C), metallodipeptidase activity was overrepresented at weeks 2 and 5; serine-type endopeptidase inhibitor activity and antigen binding were overrepresented at weeks 2 and 7.

To characterize the canonical pathways involved with these differential proteins, IPA software was used for analysis. As shown in Fig. 5D, LXR/RXR activation and FXR/RXR activation were enriched at three time points. Sphingosine and sphingosine-1-phosphate metabolism, ceramide degradation, lactose degradation III, and thyroid hormone biosynthesis were enriched at weeks 5 and 7. Complement system, sucrose degradation V, IL-12 signaling and production in macrophages, and glycolysis I were enriched at week 2. Glutamate degradation II was enriched at week 5. Aryl hydrocarbon Receptor signaling was enriched at week 7.

## Discussion

In this study, daily moderate-intensity swimming exercise rat model was established. Compared to the control group, a total of 112, 61 and 44 differential proteins were identified after 2, 5 and 7 weeks of swimming exercise, respectively. Randomized grouping statistical analysis showed that more than 85% of the differential proteins identified in this study were caused by swimming exercise rather than random allocation.

By biological process analysis, we found that some processes of differential proteins were consistent with previous researches. For example, some immune-related processes were enriched after swimming exercise. Exercise has a profound effect on immune system function, and studies have shown that regular moderate intensity exercise is beneficial for immunity (Pedersen & Hoffman-Goetz, 2000; Simpson et al., 2015). Furthermore, we found that positive regulation of cholesterol esterification was enriched after swimming exercise in this study. Regular physical exercise provides a wide range of cardiovascular benefits as a nonpharmacological treatment and promotes cholesterol esterification and transport from peripheral tissues to the liver (Simko & Kelley, 1979; Mann, Beedie & Jimenez, 2014).

Additionally, some pathways were previously reported to be associated with physical exercise. For example, sphingosine-1-phosphate (S1P) plays an important role in skeletal muscle pathophysiology, and S1P metabolism was found to be regulated by exercise (Hodun, Chabowski & Baranowski, 2021). The S1P content in plasma and its receptors in skeletal muscles were reported to be increased in the skeletal muscle of rats after resistance training (Banitalebi et al., 2013). Sphingosine and sphingosine-1-phosphate metabolism were enriched in the urine after swimming exercise in this study. Additionally, carbohydrates are the most efficient fuel for working muscles. The first metabolic pathways of carbohydrate metabolism are skeletal muscle glycogenolysis and glycolysis, and circulating glucose becomes an important energy source. Lactate was also reported to play a primary role as either a direct or indirect energy source for contracting skeletal muscle. We found that some glucose metabolism-related pathways were enriched in urine. Furthermore, glutamate has been implicated in exhaustive or vigorous exercise (Guezennec et al., 1998), and a study showed that glutamate increased significantly in the visual cortex following exercise (Maddock et al., 2016). In this study, we found that glutamate degradation II was enriched in urine following moderate-intensity exercise. Overall, the urine proteome can reflect changes associated with physical exercise.

This study was a preliminary study with a limited number of rats, and the differential proteins identified in this study require further verification in a large number of human urine samples. Urine proteomes after different lengths of exercise were different, suggesting that urine proteomics may distinguish long-term and short-term responses to exercise. Additionally, this is a starting point for further studies of urinary proteome after different types and intensities of exercise to monitor the amount of exercise and to develop an optimal exercise plan. Physical exercise may be an influencing factor in urine proteomics research. When using human urine samples to discover disease biomarkers, physical exercise-related effects can be excluded in future studies.

## Conclusions

Our results revealed that the urinary proteome could reflect significant changes after swimming exercise. These findings may provide an approach to monitor the effects of exercise of the body.

## Supplemental Information

10.7717/peerj.12406/supp-1 Supplemental Information 1 Unsupervised clustering analysis of all of proteins identified at three time points

10.7717/peerj.12406/supp-2 Supplemental Information 2 The variable isolation window of the DIA method with 36 windows was set for DIA acquisition

10.7717/peerj.12406/supp-3 Supplemental Information 3 The identification and quantification details of proteins identified in this study

10.7717/peerj.12406/supp-4 Supplemental Information 4 The results of randomized grouping statistical analysis

10.7717/peerj.12406/supp-5 Supplemental Information 5 ARRIVE 2.0 checklist

## Additional Information and Declarations

### Data Availability

The following information was supplied regarding data availability: The mass spectrometry proteomics data are available at ProteomeXchange Consortium: PXD025814.
