# Supplementary figures and images for: Changes in the urinary proteome in rats with regular swimming exercise
Source: PeerJ. 2021 Nov 1;9:e12406. doi: 10.7717/peerj.12406 (PMC8567855; doi:10.7717/peerj.12406)

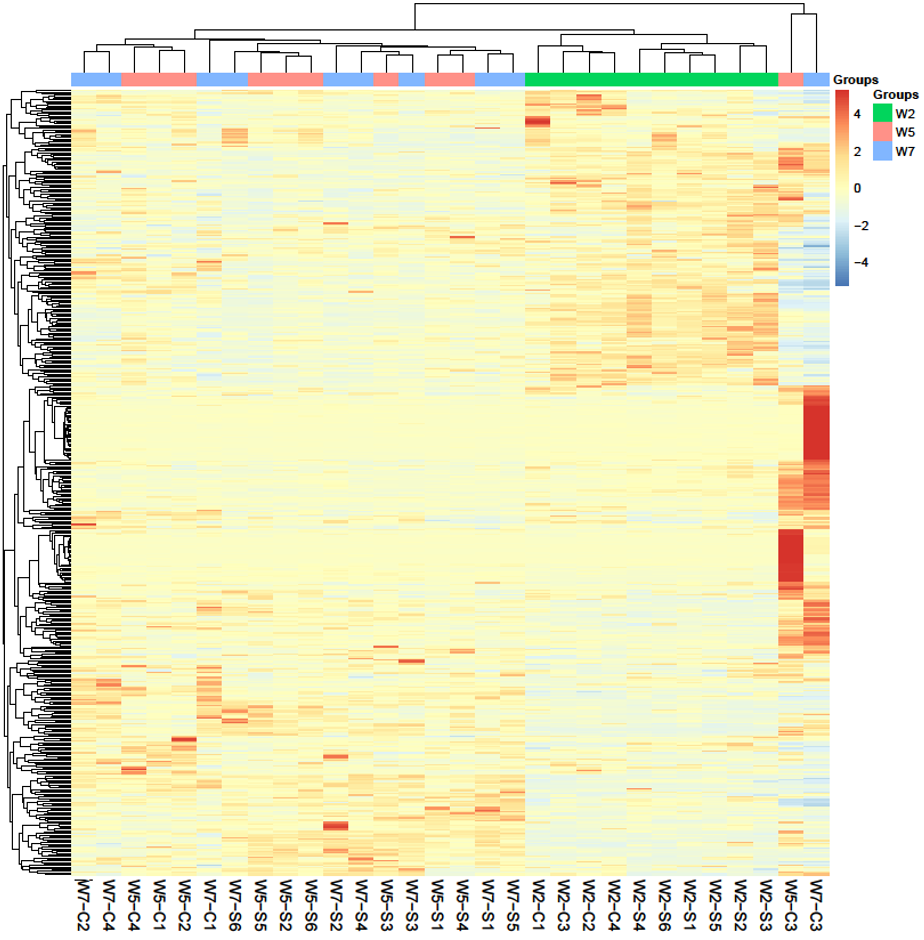

Supplement: Supplemental Information 1 [file peerj-09-12406-s001.png]
